# An Atypical Presentation of Scabies

**DOI:** 10.4269/ajtmh.21-0714

**Published:** 2021-09-04

**Authors:** Arezki Izri, Mohammad Akhoundi

**Affiliations:** ^1^ Parasitology-Mycology Department, Avicenne Hospital, AP-HP, Paris 13 University, Bobigny, France;; ^2^ Unité des Virus Émergents (UVE: Aix-Marseille Univ-IRD 190-Inserm 1207-IHU Méditerranée Infection), Marseille, France

**Figure 1.**
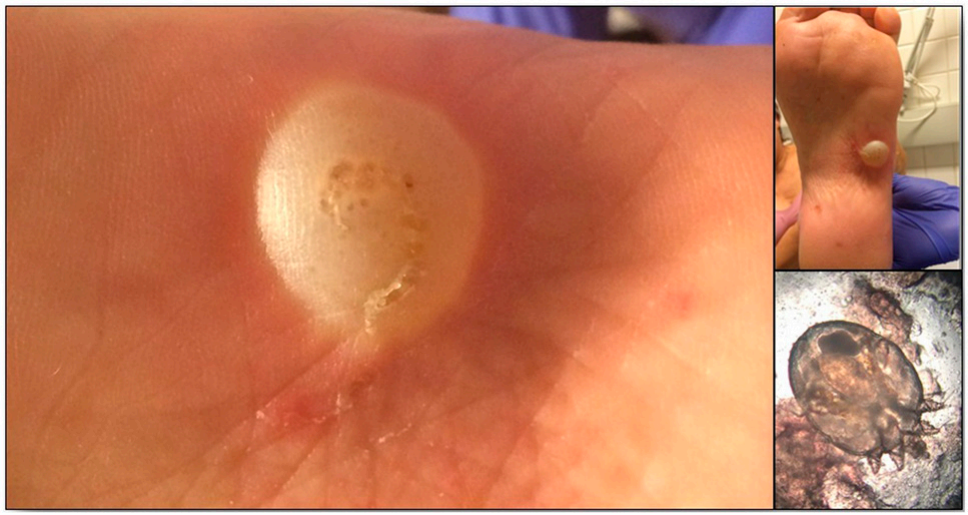
An atypical presentation of scabies. This figure appears in color at www.ajtmh.org.

A 45-year-old woman was referred for foot pain of 3 days duration that impaired walking. Clinical examinations did not reveal any skin abnormalities other than on the plantar aspect of the left foot, where a blister with a linear burrow typical of scabies. Microscopic examination of skin scrapings showed a female *Sarcoptes scabiei* mite under 100× magnification. Treatment with biseptin (antiseptic lotion) and ivermectin (200 µg/kg, in first and tenth days) led to a favorable outcome 2 weeks later. Human scabies, a skin infestation caused by *Sarcoptes scabiei* var. *hominis* mite, occurs worldwide in all ethnic groups and socioeconomic levels.^1^^,^^2^ It spreads by skin-to-skin contact.^3^ The mites are usually found between the fingers, wrists or genitals.^4^ Nevertheless, they can appear anywhere on the body and lesions can be exacerbated by immunosuppression. The infested location (plantar) and the clinical manifestation as demonstrated here are uncommon. Clinicians should be aware of such unusual clinical signs and symptoms resulting from *Sarcoptes* infestation (Figure 1).
